# Decomposing the inequalities in the catastrophic health expenditures on the hospitalization in India: empirical evidence from national sample survey data

**DOI:** 10.3389/fpubh.2024.1329447

**Published:** 2024-04-04

**Authors:** Shyamkumar Sriram, Veenapani Rajeev Verma, Pavan Kumar Gollapalli, Muayad Albadrani

**Affiliations:** ^1^Department of Social and Public Health, College of Health Sciences and Professions, Ohio University, Athens, OH, United States; ^2^Indian Institute of Technology Madras, Chennai, Tamil Nadu, India; ^3^Chettinad Hospital and Research Institute, Chennai, Tamil Nadu, India; ^4^Taibah University, Medina, Saudi Arabia

**Keywords:** out-of-pocket healthcare expenditures, hospitalization care, catastrophic health expenditures, inequality, need-adjusted inequities, decomposition of inequality

## Abstract

**Introduction:**

Sustainable Development Goal (SDG) Target 3.8.2 entails financial protection against catastrophic health expenditure (CHE) by reducing out-of-pocket expenditure (OOPE) on healthcare. India is characterized by one of the highest OOPE on healthcare, in conjunction with the pervasive socio-economic disparities entrenched in the population. As a corollary, India has embarked on the trajectory of ensuring financial risk protection, particularly for the poor, with the launch of various flagship initiatives. Overall, the evidence on wealth-related inequities in the incidence of CHE in low- and middle-Income countries has been heterogenous. Thus, this study was conducted to estimate the income-related inequalities in the incidence of CHE on hospitalization and glean the individual contributions of wider socio-economic determinants in influencing these inequalities in India.

**Methods:**

The study employed cross-sectional data from the nationally represented survey on morbidity and healthcare (75th round of National Sample Survey Organization) conducted during 2017–2018, which circumscribed a sample size of 1,13,823 households and 5,57,887 individuals. The inequalities and need-adjusted inequities in the incidence of CHE on hospitalization care were assessed via the Erreygers corrected concentration index. Need-standardized concentration indices were further used to unravel the inter- and intra-regional income-related inequities in the outcome of interest. The factors associated with the incidence of CHE were explored using multivariate logistic regression within the framework of Andersen’s model of behavioral health. Additionally, regression-based decomposition was performed to delineate the individual contributions of legitimate and illegitimate factors in the measured inequalities of CHE.

**Results:**

Our findings revealed pervasive wealth-related inequalities in the CHE for hospitalization care in India, with a profound gap between the poorest and richest income quintiles. The negative value of the concentration index (EI: −0.19) indicated that the inequalities were significantly concentrated among the poor. Furthermore, the need-adjusted inequalities also demonstrated the pro-poor concentration (EI: −0.26), denoting the unfair systemic inequalities in the CHE, which are disadvantageous to the poor. Multivariate logistic results indicated that households with older adult, smaller size, vulnerable caste affiliation, poorest income quintile, no insurance cover, hospitalization in a private facility, longer stay duration in the hospital, and residence in the region at a lower level of epidemiological transition level were associated with increased likelihood of incurring CHE on hospitalization. The decomposition analysis unraveled that the contribution of non-need/illegitimate factors (127.1%) in driving the inequality was positive and relatively high vis-à-vis negative low contribution of need/legitimate factors (35.3%). However, most of the unfair inequalities were accounted for by socio-structural factors such as the size of the household and enabling factors such as income group and utilization pattern.

**Conclusion:**

The study underscored the skewed distribution of CHE as the poor were found to incur more CHE on hospitalization care despite the targeted programs by the government. Concomitantly, most of the inequality was driven by illegitimate factors amenable to policy change. Thus, policy interventions such as increasing the awareness, enrollment, and utilization of Publicly Financed Health Insurance schemes, strengthening the public hospitals to provide improved quality of specialized care and referral mechanisms, and increasing the overall budgetary share of healthcare to improve the institutional capacities are suggested.

## 1 Introduction

The Universal Health Coverage (UHC) has been proclaimed as the third major transition in health, after the demographic and epidemiological transitions (1) and has become the focal point of health policy discourse as the world made transition from millennium development goals (MDSs) to sustainable development goals (SDGs). Goal 3.8 of the SDG Agenda enunciates to achieve the UHC and encompasses two components: (i) Indicator 3.8.1–Coverage of essential health services (defined as average coverage of essential services based on tracer interventions that include reproductive, maternal, newborn, and child health, infectious diseases, non-communicable diseases, and service capacity and access, among the general and most disadvantaged population). (ii) Indicator 3.8.2–Incidence of catastrophic health spending (defined as the proportion of the population with large household expenditures on health as a share of total household expenditure or income). Despite the institutional commitment, there is an inordinate reliance on out-of-pocket-expenditure (OOPE) to finance healthcare due to the severely underfunded health system. For India, specifically, the public health expenditure as a share of GDP (1.25%) is the lowest in the world. Furthermore, the estimates from the National Health Accounts of India divulged that abysmally low coverage of government-sponsored pre-payment schemes coupled with the dearth of private health insurance has impelled households to have excessive reliance on out-of-pocket payments (58.7% of total health expenditure) for healthcare (2).

Healthcare expenditures or costs are incurred whenever a person accesses the healthcare system and utilizes the healthcare services. Health expenditures could be broadly defined as any expense that is spent on healthcare and related activities, including paying premiums for private or public health insurance coverage (3). A multitude of cost components encompasses healthcare payments on hospitalization, such as direct medical costs related to user fees, made at the time of health service use, incorporating charges ranging from registration, consultation, drugs, diagnostics, bed charges, etc. A legion of studies examining the impact of user fees on healthcare-seeking behavior in LMICs have conceded that the higher user fee/increase in prices can lead to decreased healthcare utilization and *vice-versa* (4–6). Literature in the Indian context underscores the impact of user charges and direct medical costs, specifically on drugs and diagnostics (7, 8). In addition to the direct cost, indirect costs, such as expenses on food, lodging, and transportation, also account for a large proportion of OOPE, as evinced in the literature from LMICs and India (9–13). Furthermore, other invisible costs that were not incurred because of medical management of disease but rather of other incurred losses, such as lost wages, lost productivity, and costs resulting from the need for home care and child care otherwise not incurred, also pose a formidable barrier to access.

The unprecedented level of financial burden posed by healthcare expenditures has two-pronged implications. First, at the macroeconomic level, the burden posed by forgone care due to affordability barriers has a deleterious impact on the economic growth of the region due to loss in productivity. Second, out-of-pocket health payments precipitate an adverse shock on the financial stability of households incurring such expenditure, subsequently rendering the households vulnerable to catastrophic health expenditure and impoverishment due to income shocks perpetuated via health shocks, which can further potentially culminate into a trans-generational cycle of poverty, bearing long-term consequences. Health shock is the most common idiosyncratic income shock and one of the most pertinent reasons for the descent of households into poverty in LMICs (14).

The out-of-pocket payments for healthcare are usually the most inequitable type of finance due to its tendency to hit the poor the hardest by being a barrier to healthcare/by denying individuals’ financial protection from catastrophic illness (15). Studies from India have established the Inverse Care Law, i.e., individuals with the greatest need for healthcare have the greatest difficulty in accessing healthcare services (16–18). There is strong evidence that financial access to healthcare is very low among those residing in rural areas, uneducated, lowest wealth quintile, and otherwise marginalized sections of society (19). In a resource-poor setting, there are substantial heterogeneities in healthcare measures and capacity to pay thereof; as a corollary, pervasive income-based inequalities in the economic burden of care on the households are pronounced in these settings as well. A systematic review of LMICs has evinced that across all the LMICs, the risk of incurring CHE is six times more concentrated among the poor (20). Furthermore, evidence on hospitalization from countries such as Argentina, China, India, and Tanzania also revealed the disproportionate impact of CHE on the poor (21). Although there is some literature on the impact of socio-economic inequalities on the incidence of catastrophic payments in the Indian context (22–24), the evidence is rather exiguous and does not commensurate with the policy implications.

In India, the National Health Policy 2017 (25) directed that budgetary allocations would ensure horizontal equity by targeting specific population subgroups, geographical areas, healthcare services, and gender-related issues. Horizontal equity entails equal treatment for equal needs, irrespective of other socio-economic characteristics such as income, education, place of residence, and social group. Meanwhile, vertical equity connotes unequal treatment for unequal needs. However, the measurement of horizontal inequities is quite complex vis-a-vis vertical inequality, as need is a rather elusive concept both in terms of the choice of measurable indicators and also normative ethical considerations (26). However, the degree to which health inequality is considered inequitable is estimated via the need-adjustment of inequality. Literature commonly suggests that people with similar health statuses have the same needs and persons with dissimilar health statuses have different needs (27). The need-based variables are not amenable to the policy intervention and, thus, considered as fair or legitimate variables, whereas non-need variables are due to systemic inequalities and are amenable to policy intervention, thus, considered as unfair or illegitimate. Therefore, standardizing the inequality in health outcomes by need results in systematic disparities and captures the degree to which the inequality is inequitable.

The systemic inequalities along the socio-economic gradient with respect to the burden of healthcare payments continue to pose an unprecedented challenge in India despite the launch of various initiatives to provide financial risk protection to the poor and vulnerable. Previous studies have revealed that the incidence of CHE on hospitalization care has increased in the last few decades in India (24). However, the evidence of the impact of these initiatives in reducing the catastrophic burden among poor households remains elusive. Thus, it becomes imperative to explore the dimension of equity w.r.t. incidence of the catastrophic burden of out-of-pocket payments to correct existing interventions and promulgate inclusive policies.

However, there is a dearth of literature to study the need-adjusted inequities in the incidence of CHE for hospitalization care, and, further, to the best of our knowledge, no study has been conducted to decompose the effect of the legitimate and illegitimate factors causing the inequalities in the CHE. At the same time, it is pertinent to decompose and identify the need and non-need factors that affect the health and financial protection in the household to enable the targeted policy response. Thus, this study was conducted to estimate the degree of inequalities and need-adjusted inequities in the incidence of CHE for hospitalization care using a modified Erreygers concentration index. Furthermore, wider socio-economic-contextual determinates influencing the CHE on hospitalization care were unraveled succinctly within a conceptual framework. Additionally, the study also attempted to measure the relative contributions of need and non-need factors driving the inequality in the CHE by conducting a robust regression-based decomposition of the inequalities to identify the key variables for the policy response.

## 2 Methods

### 2.1 Data

The study employed national representative unit-level cross-sectional data from the 75th round of the *National Sample Survey Organization (Household Social Consumption in India: Health)*. The survey was conducted under the stewardship of the *Ministry of Statistics and Program Implementation*, Government of India, during the time period of July 2017–June 2018. The survey schedule collects information pertaining to the *demographic*-*socio-economic characteristics*, *morbidity status*, *utilization* of *healthcare services,* and *healthcare expenditure* across *ambulatory, inpatient, delivery, and immunization* care for households and individuals. A two-stage stratified random sampling design was adopted in the survey with census villages and urban blocks as the First Stage Units for rural and urban areas, respectively, and households as the Second Stage Units. The overall sample size consisted of 1,13,823 households and 5,57,887 individuals (including the death cases). The analysis, however, circumscribed 66,237 individuals who were hospitalized in the last 365 days of the survey (without childbirth episodes). For this study, the information encompassing both medical expenses such as doctor’s/surgeon’s fee, medicines, diagnostic tests, bed charges, and consumables, *viz.* blood, oxygen, etc., and non-medical expenses such as expenses incurred on transportation, food, and lodging on account of treatment was employed in the study. Detailed information on the survey design can be found in the official report released by the National Sample Survey Organization (28).

### 2.2 Measures

The following measures were assessed in the study: (a) Extent of CHE on hospitalization cases in India; (b) Wealth-related inequities in the incidence of CHE on hospitalization; (c) Socio-economic-demographic factors impacting the CHE on hospitalization cases; and (d) Relative contribution of the factors in driving the wealth-based inequality in the CHE for hospitalization cases.

#### 2.2.1 Outcome measure

The survey encompasses information on the expenses incurred in hospital treatment (medical and non-medical). The medical component subsumed data on the expenses toward the doctor’s/surgeon’s fee, medicines, diagnostics, bed charges, physiotherapy, personal medical appliances, and other consumables such as oxygen and blood. However, the non-medical component incorporated the expenses incurred on other ancillary payments, such as transportation, lodging, and food for the patient and caretaker, on account of the treatment. Given the information, the out-of-pocket expenditure (OOPE) is then defined as the direct payments made by the patients at the time of treatment, net of any reimbursements by the insurance provider. The CHE can be defined via two approaches, i.e., (a) capacity-to-pay approach and (b) budget-share approach. Under the capacity-to-pay approach, the OOPE on healthcare is considered catastrophic if a household’s financial contributions to the healthcare treatment exceed the 40% of income remaining after the subsistence needs have been met (29, 30). Meanwhile, under the Budget-share approach, the OOPE is catastrophic if a household’s financial contribution to the treatment equals or exceeds 10% of the household’s total expenditure (31, 32). In this study, the CHE was computed using the budget-share approach, where a 10% threshold of total household expenditure was considered. The outcome variable of interest in the study was binary in nature, indicating whether a household faced CHE on inpatient treatment.

#### 2.2.2 Covariates

A gamut of household and individual level variables, drawn from Andersen’s *behavioral health model* (33), were incorporated into the study. The covariates were cogitated into legitimate/need and illegitimate/non-need variables to unravel the horizontal inequities underlying the CHE. The need for healthcare is considered an elusive concept, and the choice of variables is embedded in the normative categorization, which requires a potentially contestable value judgment (27). In general, the need sources of variation in health are ethically acceptable, whereas the non-need sources are ethically unjust or unfair (34). The variables underscoring the differential need for healthcare expenditure, *viz.* demographic characteristics, health status, and severity of ailments, such as age composition of household members, number of chronic members, hospitalization cases in households, and duration of stay in the hospital, were considered as the need-based variables in the study.

A myriad of factors impacted the choice of non-need variables, such as previous literature (35–37), relevance to explaining the inequality within the available dataset, and availability of periodic and routine monitoring of the indicators. A broad spectrum of household-level variables across the *demographic characteristics* such as age and gender of the household head, household size, and marital status of the household members; *socio-economic characteristics,* such as education, social group, religion, principal occupation of the household, monthly household consumption expenditure, and housing conditions (comprehensive indicator coalescing information on the drinking water source, cooking source, drainage type, and garbage disposal)*; enabling characteristics,* such as insurance coverage and type of facility where care is sought; and *contextual* var*iables* such as the level of epidemiological transition level of the residential region and the geographical location (urban/rural) were chosen as the non-need variables. The monthly household consumption expenditure was adjusted to account for the economies of scale in household consumption stemming from the household size and demographic composition due to underlying differences in need among the household members using the Oxford equivalence scale (38). Furthermore, the monthly consumption household expenditure was converted to the annual expenditure to make it uniform with the expenses incurred on hospitalization with a recall period of 365 days.

### 2.3 Statistical analysis

#### 2.3.1 Incidence of catastrophic health expenditure

The incidence of catastrophic health expenditure was computed via a budget-share approach and elucidated as the share of out-of-pocket health expenditure and out of the total household expenditure:

1 $$S_{i}=\frac{OOPE_{i}}{THE_{i}}$$

Where, $OOPE_{i}$ is the out-of-pocket expenditure of household i , $THE_{i}$ is the household’s total consumption expenditure of household i , and $S_{i}$ is the share of the total healthcare expenditure out of the total consumption expenditure of household i . Consider $Z_{i}$ is the threshold beyond which the household i incurs catastrophic expenditure if $S_{i}>10\%$ , which can be represented as:


$$Z_{i}\;=1\;\mathrm{if}\;S_{i}>10\%$$

and


$$Z_{i}\;=0\;\mathrm{if}\;S_{i}>10\%$$

#### 2.3.2 Concentration curve and index

The concentration curve was used to glean the inequities in the CHE on hospitalization care. Cumulative proportions of the catastrophic health payment (vertical axis) were plotted against the cumulative proportion of the households with hospitalization cases (horizontal axis), ranked by the equivalized household consumption expenditure. The concentration index, denoted by C, is estimated as twice the area between the concentration curve and diagonal, which is represented as:


2 $$CI=\frac{2}{\eta \mu }\overset{n}{\underset{i=1}{\sum }}CHE_{i}R_{i}-1$$

where, $CHE_{i}$ is the variable of interest for the household; μ is the mean of $CHE_{i}$ ; and $R_{i}$ is the $i^{th}$ ranked household in the socio-economic distribution from most disadvantaged (i.e., poorest) to the least disadvantaged (i.e., richest). The value of CI ranges between −1 and + 1, where a positive value indicates the distribution concentrated among the rich and a negative value represents a distribution concentrated among the poor.

#### 2.3.3 Choice of index

The outcome variable chosen in our study is binary, which is not consonant with the standard concentration index that measures relative inequality and does not allow for the differences between the individuals to be compared. When the standard concentration index is applied to the binary variable, characterized by ordinal and bounded nature, erroneous estimates are produced due to the following reasons: (a) An increase in the binary measure is mirrored by the decrease in the measure; (b) An equi-proportionate increase in the binary measure does not translate to the equi-proportionate decrease in the measure; and (c) Bounds act as constraints to (proportionally) equal transformations of the binary measure. The standard concentration index violates the mirror condition and cardinal invariance property. Additionally, a scale-invariant and rank-dependent index, such as the standard concentration index, fails to account for mirror conditions while accounting for the relative differences simultaneously (39, 40). These conditions, however, can be satisfied by the generalized version of the modified concentration index proposed by Wagstaff (41) or Erreygers corrected concentration index (39). The generalized concentration index departs from the Erreygers index based on value judgments related to the desirability of level independence (42). This study employed the Erreygers corrected concentration index to compute the wealth-related inequalities in incurring the CHE by the households. Erreygers corrected concentration index is an absolute rather than a relative measure and is only a rank-dependent measure, which is suitable for our binary outcome measure as it satisfies all the desirable properties for rank-dependent indices, i.e., mirror, transfer, cardinal invariance, and level independence. Furthermore, Erreygers has developed the notions of ‘quasi-absoluteness’ and ‘quasi-relativity’ best suited for the bounded variables as they mitigate the infeasibility of equi-proportional change or equal additions in binary constructs. The index is represented as:


3 $$EI=\frac{4\mu }{\left(\;b_{n}-a_{n}\right)}\;CI$$

Where CI denotes the standard concentration index as represented in Equation 2, μ is the mean of CHE in the population, and $a_{n}$ , $b_{n}$ are the upper and lower bounds of the outcome variables.

#### 2.3.4 Need standardization

The differential role of need-based factors such as health conditions and demographics in driving health inequality is not considered in the unstandardized distribution of the outcome measures. However, the differential role of such factors can be observed by segregating the inequality into legitimate and illegitimate health inequality. As a result, the need-standardization was conducted to adjust for the legitimate factors impacting health inequality and to facilitate the comparison across groups. The need-standardization can be done via direct-standardization and indirect-standardization methods. The indirect standardization, reflecting the actual distribution of healthcare outcomes and the distribution that would be expected given the distribution of need, was adopted in this study. The indirect standardization exhibits greater accuracy when dealing with unit-level data. However, the evidence on standardization of equity procedures suggests that inequity measures do not digress significantly with the use of linear methods vis-a-vis non-linear methods (43, 44). Thus, a linear regression model for standardization was employed first, which is depicted as follows:


4 $$y_{i}=\alpha +\underset{n}{\sum }\beta_{j}x_{ji}+\underset{m}{\sum }\theta_{k}Z_{ki}+\varepsilon_{i}$$

Where, $y_{i}$ is the CHE for the household i ; $x_{ji}$ and $Z_{ki}$ are the vectors of need and non-need factors driving the inequality; α , $\beta_{j}\text{,}$ and $\theta_{k}$ are the parameters, while the $\varepsilon_{i}$ is the error term. Additionally, the predicted values of the outcome measure ( ${{\hat{y}}_{i}}^{x}$ ) was obtained using the OLS parameter estimates ( $\hat{a}$ , ${\hat{\beta }}_{j}$ , and ${\hat{\theta }}_{k}$ ), individual values of the need-variables ( $x_{ji}$ ), and sampled means of the controlled non-need variables ( ${\bar{z}}_{ji}$ ). In the next step, the estimates for indirect standardization of outcome measure ( ${{\hat{y}}_{i}}^{\mathrm{IS}}$ ) was obtained by subtracting the predicted values from actual values and adding the overall sample mean ( $\bar{y}$ ). The subsequent procedure is depicted as follows:


5 $${{\hat{y}}_{i}}^{\mathrm{IS}}=y_{i}-{{\hat{y}}_{i}}^{x}+\bar{y}$$

#### 2.3.5 Decomposition of concentration index

The Erreygers concentration index was decomposed to estimate the relative contribution of covariates to explain the inequality in the outcome measure and other unexplained residual variations. A linear approximation of the model, which is based on the partial effects of each covariate evaluated at the sample means, was employed to perform the decomposition. The linear decomposition of inequalities in outcome measure is illustrated as:


6 $$CI=\underset{m}{\sum }\left(\frac{\beta_{j}{\bar{x}}_{j}}{\bar{y}}\right)\;CI_{j}+\underset{n}{\sum }\left(\frac{\gamma_{k}{\bar{z}}_{k}}{\bar{y}}\right)\;CI_{k}+\frac{GCI_{\varepsilon }}{\bar{y}}$$

Where, ${\bar{x}}_{j}$ and ${\bar{z}}_{j}$ denotes the means of need and non-need factors, respectively, whereas, $CI_{j}$ and $CI_{k}$ are representative of the respective concentration indices. $GCI_{\varepsilon }$ is the generalized concentration index for $\varepsilon_{i}$ (residual term), which corresponds to the inequality in the outcome measure that cannot be explained by the systematic variation in other variables. The representation is depicted below:


7 $$GCI_{\varepsilon }=\frac{2}{n}\;\overset{n}{\underset{i=1}{\sum }}\varepsilon_{i}R_{i}$$

The modified form of decomposition of Erreyger’s index is thus, given as (44):


8 $$EI_{c}=4\underset{m}{\sum }\left(\beta_{j}{\bar{x}}_{j}\right)\;CI_{j}+\underset{n}{\sum }\left(\gamma_{k}{\bar{z}}_{k}\right)CI_{k}+GCI_{\varepsilon }$$

The horizontal inequity (HI) in the CHE was thus estimated by subtracting the absolute contributions made by the need-based factors from the unadjusted value of the Erreygers index. A positive value of HI indicates the inequities concentrated among the better-off, whereas a negative value indicates the inequities concentrated among the worse-off.

#### 2.3.6 Determinants of catastrophic health expenditure

The determinants of CHE were gleaned using a gamut of variables that were embedded within Andersen’s behavioral health model (45). As per the Andersen framework, the choice variables were prorated into (a) *Predisposing components* reflecting the demographic and socio-structural characteristics of the household; (b) *Enabling components* subsuming standard of living and insurance coverage for the households; (c) *Need components* underscoring the severity of disease, frequency, and duration of hospitalization episodes; and (d) *Contextual components* comprising the regional aspects such as spatial location and burden of the NCD’s in the region.

A multivariate logistic regression model was employed to unravel the determinants of CHE, represented as:


9 $$S_{i}=\ln\left(\frac{\hat{y}}{1-\hat{y}}\right)=\beta_{0}+\beta_{1}X_{1}+\beta_{2}X_{2}+\ldots \ldots .\beta_{n}X_{n}$$

where, the $S_{i}$ , which is the share of out-of-pocket health expenditure ( $OOPE_{i}$ ) out of the total health expenditure ( $THE_{i}$ ), is dichotomous, i.e., $S_{i}$ assumes the value of 1 if the out-of-pocket health expenditure ( $OOPE_{i}$ ) exceeds the 10% threshold of the total health expenditure $\left(THE_{i}\right)$ and 0 otherwise. The notation $X_{1\text{,}}$ $X_{2}$ ….. $X_{n}$ represents the socio-economic-demographic-contextual variables driving the CHE. The analysis was conducted using the STATA 15.0 statistical package. The estimates were weighted to account for the complex multistage sample design and confidence intervals for the horizontal inequity index were computed using Bootstrap with 1,000 replications.

## 3 Results

**Figure 1:**
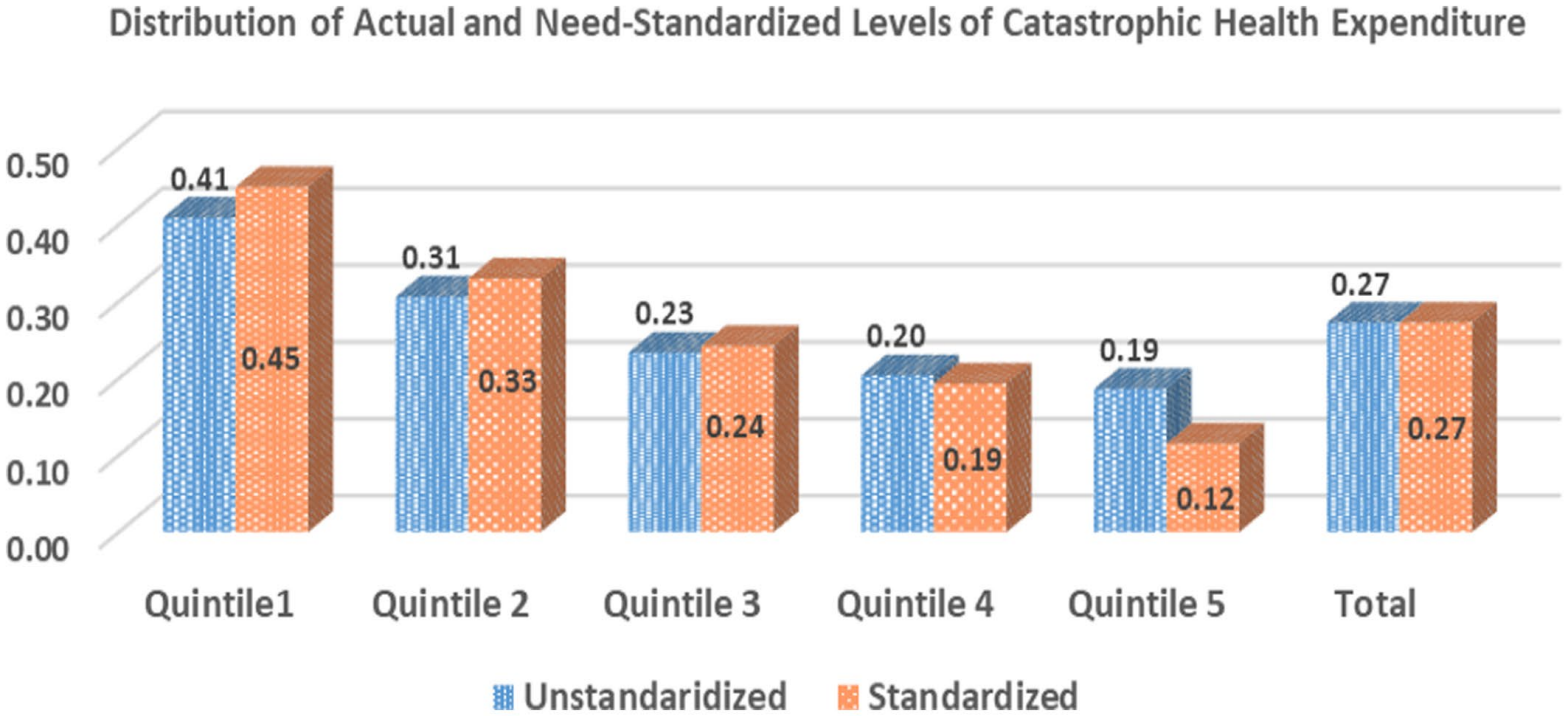
Distribution of actual and need-standardized levels of CHE on inpatient care in India.

The unstandardized and need-standardized distribution of CHE on Hospitalization care in India is illustrated in Figure 1. Overall, 27% of the ailing treated as inpatients (except for childbirth) incurred CHE during 2017–2018 in India. The incidence of CHE, however, exhibited an inverse relationship with the relative ranking of the expenditure quintile groups. An extensive gradient in the levels of CHE was found between the lowest and highest quintile groups. The incidence of CHE for the population hospitalized in the poorest quintile (41%) was more than twice as compared to the richest quintile (19%). Furthermore, the estimates of the need-standardized CHE were found to be higher than the unstandardized CHE estimates for poor- and middle-income groups (need-standardized CHE greater than unstandardized by 4, 2, and 1% points for poorest, poor, and middle quintile groups); whereas, standardized CHE levels were less than the unstandardized estimates for the wealthier groups (need-standardized CHE lesser than unstandardized estimates by 1 and 7% for rich and richest quintile groups, respectively).

### 3.1 Inequality and inequities in the catastrophic health expenditure on hospitalization care

**Figure 2:**
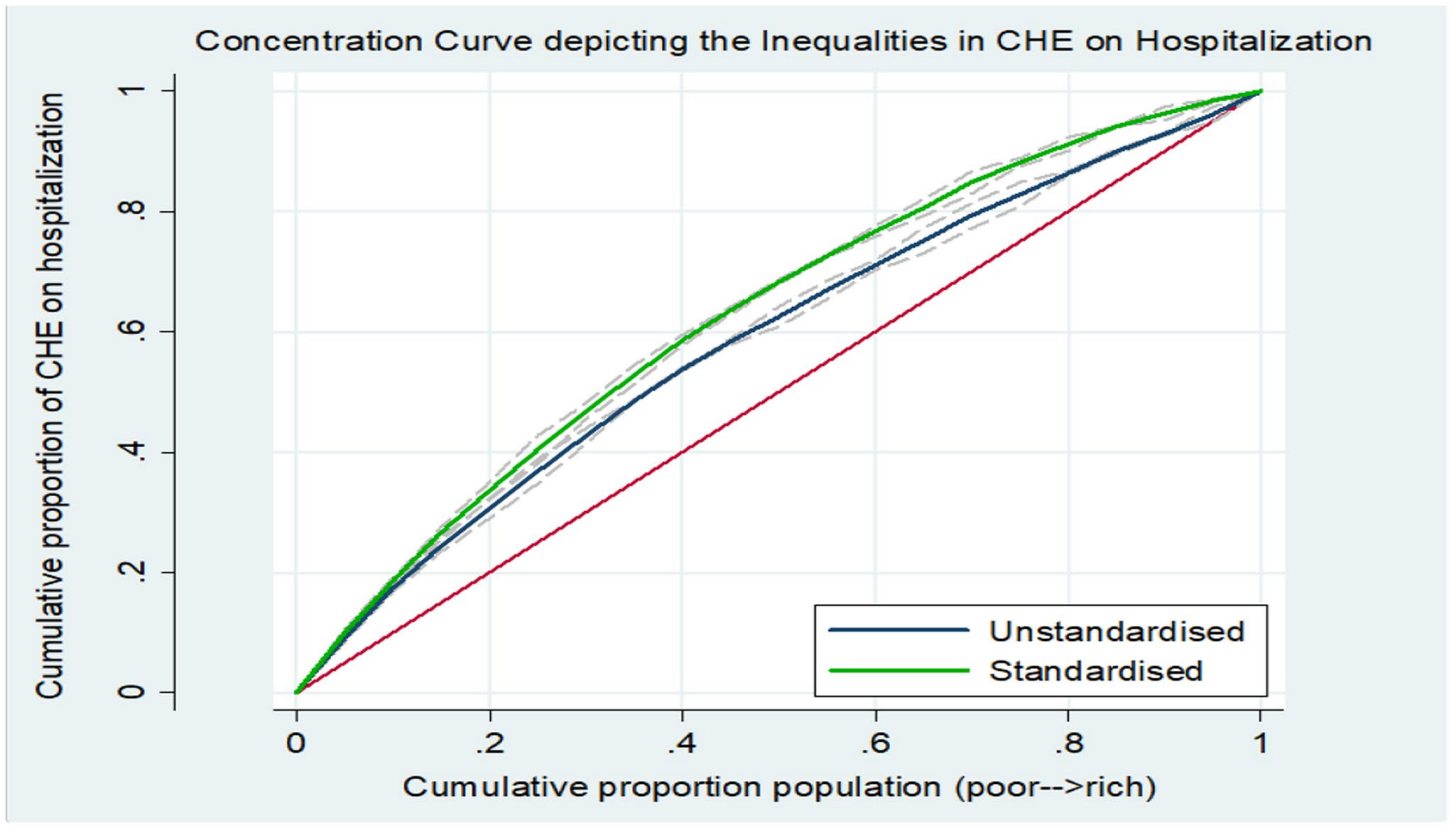
Concentration curves depicting the inequalities in CHE on inpatient care in India.

The concentration curve eliciting the inequalities and inequities in the CHE on hospitalization care is plotted in Figure 2. The concentration curve (unstandardized) was found to be above (dominates) the line of equality, indicating that the burden of CHE on inpatient care was concentrated among the poor. Furthermore, the standardized curve (adjusted for differential needs) dominated the unstandardized curve, which denoted that for equal need, the concentration of inequality among the poor was more pronounced vis-a-vis the inequality in CHE, which is not adjusted by the need-based confounding factors. The dominance testing to test the difference between estimated concentration curve ordinates and diagonal via the Multiple Comparison Approach and Intersection Union Principle rejected the null of no wealth-related inequality and established that concentration curves significantly dominated the line of equality. Correspondingly, the estimated value of the Erreyger’s corrected concentration index (Table 1) was negative and significant (EI: -0.191; *p* < 0.05), underscoring the disproportionate incidence of CHE among the poor in India. Moreover, the estimates of the need-adjusted concentration index (EI: -0.258; *p* < 0.01) corroborated the wider inequities when accounting for the differential needs.

**Table 1:** Concentration indices depicting the inequality in CHE for hospitalization care.

	Index values	Standard error	*p*-value
Erreyger’s concentration index	–0.191**	0.0151	0.050
Need-adjusted index	–0.258***	0.0021	0.009

Level of significance: ***p < 0.01; **p < 0.05; *p < 0.1.

### 3.2 Inter-state differentials in the inequities in CHE on hospitalization

**Figure 3:**
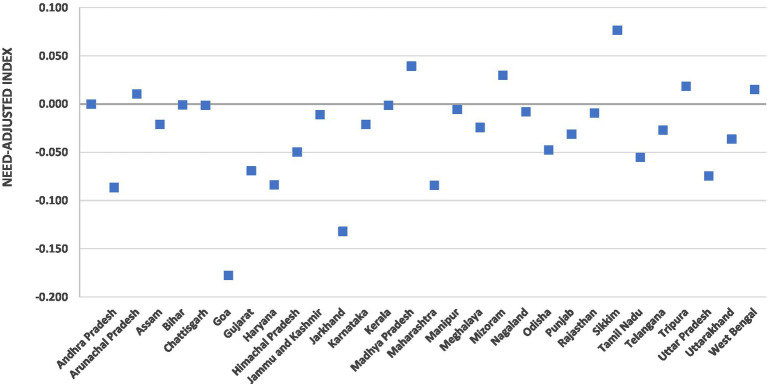
Need-adjusted inequality indices for CHE on hospitalization in Indian states.

The extent of the need-adjusted wealth inequities in incurring the CHE on inpatient care is exhibited in Figure 3. The measure of inequity was perceptibly concentrated among the poor in most of the Indian states. However, substantial heterogeneities were found in the degree of the inequities among the states. Wealth-related inequities (concentrated among the poor) were found to be high in the states such as Goa (EI: −0.18) and Jharkhand (EI: −0.13). A few states, such as Uttar Pradesh and Maharashtra, with just approximately one-fourth of the total health spending financed by the government, also exhibited significantly high inequities concentrated among the poor. Conversely, no inequities (EI: 0.00) were estimated for the states of Bihar, Chhattisgarh, and Kerala. Furthermore, the states of Assam and Jammu and Kashmir with the highest level of government spending as a proportion of total health spending (55.2 and 51.3% for Assam and Jammu and Kashmir, respectively) evinced relatively less wealth-related inequities. However, the need-adjusted inequalities were concentrated among the rich in the North-Eastern states of Sikkim (EI: 0.07) and Manipur (0.03) in India.

### 3.3 Descriptive statistics of the variables

The descriptive statistics of the households with hospitalization episodes in the survey period are presented in Table 2. Most households were headed by adults aged 25–75 years (95.6%) and were men (88.6%). The demographic structure consisted of small (47.5%) and middle (50.2%)-sized households, and more than half of the households (53.5%) lived with children and older adult dependents. Furthermore, one-fourth of the households had a vulnerable widowed population. Approximately 24% of households were headed by household heads who were not literate, and a majority of the households were not employed in activities with regular sources of income. Most of the targeted surveyed households prescribed the religion of Hinduism (75.8%), followed by Islam (13.6%). Socially, a vast proportion of households belonged to the marginal communities, *viz.* scheduled caste/scheduled tribes (27.9%) and other backward castes (40.2%). Additionally, the housing conditions for most of the households were good (82.3%). However, the access to healthcare services for the household members was considerably low as more than three-fourths of the households were bereft of insurance coverage. Government-sponsored insurance coverage (14%) constituted the highest financial risk protection cover, followed by employer-sponsored coverage (4.4%). Health-seeking behavior divulged that a colossal 50.8% of households sought care from only private facilities, whereas less than half of the households (43.1%) sought care from only public facilities (43.1%). The need for healthcare was more for certain households, as approximately one-fourth of households had at least two or more members suffering from chronic ailments and had more than one hospitalization episode. The majority of the households (63.2%) accounted for a total duration of ≤7 days stay in the hospital, while 32% of households reported a hospital stay of between 7 and 14 days. Spatially, approximately 50.9% of households were residing in the states/UT’s with a higher-middle and high epidemiological transition level. Furthermore, 55.7% of households were in rural areas, while 44.3% of sampled households were residing in urban areas.

**Table 2:** Descriptive statistics of the variables.

	Variables	Percentage	C.I.
Age of household head	Below 25 years	1.2	[1.1–1.3]
	25–39 years	21.0	[20.7–21.3]
	40–59 years	53.0	[52.5–53.3]
	60–75 years	21.6	[21.3–21.9]
	Above 75 years	3.4	[3.2–3.5]
Household age composition	With both children and older people	9.8	[9.6–10.0]
	With children but no older people	18.6	[18.3–18.9]
	With older people but no children	21.9	[21.6–22.2]
	Older people only	3.2	[3.1–3.4]
	No children or old	46.5	[46.1–46.9]
Gender of household head	Female	11.4	[11.1–11.6]
	Male	88.6	[88.4–88.9]
Size of household	1–4 members	47.5	[47.1–47.9]
	5–10 members	50.2	[49.8–50.5]
	Greater than 10 members	2.3	[2.2–2.4]
Number of widows	No widow	76.2	[75.9–76.6]
	One widow	22.2	[21.9–22.5]
	Two or more widows	1.5	[1.4–1.6]
Household head education	Illiterate	23.9	[23.5–24.2]
	Literate	76.1	[75.8–76.4]
Principal activity of household	Unpaid worker/Unemployed	61.5	[61.1–61.9]
	Self employed	13.7	[13.4–14.0]
	Casual wage laborer	8.0	[7.8–8.2]
	Regular/Salaried wage employee	6.7	[6.5–6.9]
	Pensioner/Retirees	6.3	[6.1–6.5]
Social group	Scheduled tribe/Caste	27.9	[27.6–28.3]
	Other backward Caste	40.2	[39.8–40.6]
	Others	31.8	[31.5–32.2]
Religious affiliation	Hinduism	75.8	[75.5–76.1]
	Islam	13.6	[13.2–13.8]
	Christianity	6.4	[6.2–6.6]
	Others	4.2	[4.0–4.3]
Expenditure quintile groups	Poorest	20.1	[19.8–20.4]
	Poor	20.9	[20.6–21.2]
	Middle	19.1	[18.8–19.4]
	Rich	20.2	[19.9–20.5]
	Richest	19.7	[19.4–20.0]
Housing conditions	Poor	17.7	[17.4–17.9]
	Good	82.3	[82.0–82.6]
Insurance coverage	No insurance cover	78.4	[78.1–78.7]
	Government-sponsored cover	14.0	[13.8–14.2]
	Employer-sponsored cover	4.4	[4.2–4.5]
	Private insurance/Other cover	3.2	[3.0–3.3]
Type of facility	Only public	43.1	[42.7–43.5]
	Both public and private	6.1	[5.9–6.3]
	Only private	50.8	[50.4–51.1]
Members with chronic ailments	No member with chronic ailment	76.3	[76.0–76.6]
	2 members with chronic ailment	22.6	[22.3–22.9]
	More than 3 members with chronic ailment	1.1	[1.0–1.2]
Hospitalization episodes	One episode	75.3	[74.9–75.6]
	Two episodes	15.6	[15.3–15.8]
	Three-five episodes	8.6	[8.3–8.8]
	More than 5 episodes	0.6	[0.57–0.69]
Duration of stay in hospitals	1–3 days	31.8	[31.5–32.2]
	4–7 days	31.4	[31.1–31.8]
	8–14 days	32.0	[31.6–32.3]
	More than two weeks	4.7	[4.6–4.9]
Epidemiological transition level	Low epidemiological level	32.9	[32.5–33.2]
	Lower-middle epidemiological level	12.6	[12.3–12.9]
	Higher-middle epidemiological level	33.8	[33.4–34.1]
	High epidemiological level	17.1	[16.8–17.3]
Sector	Rural	55.7	[55.3–56.0]
	Urban	44.3	[44.0–44.7]

### 3.4 Factors impacting the CHE on hospitalization care among households

The wider socio-economic-contextual predictors of the CHE on hospitalization care among households in India are presented in Table 3. The estimates revealed that among the predisposing demographic factors, the age mix in the household significantly impacted the CHE. Households that were composed of only older adult members and older adult, but no children, were 9% (significant at 1% level) and 4.7% (significant at 1% level), respectively, more likely to incur the CHE vis-a-vis households with a mixed composition of both children and older adult. The structural factor of household size strongly influenced the outcome, as smaller households with less than 5 members and 5–10 members had 16.3 and 10.7%, respectively, more probability than larger households to get impacted by the CHE on inpatient care. Additionally, those households that are principally unemployed/engaged in unpaid work were less likely to be subjected to the CHE vis-a-vis households that were self-employed or receiving pensions post-retirement. Among the social characteristics, households that are ascribed to the other backward castes were more likely to suffer the catastrophic impacts of health payments compared to the households that are classified as scheduled caste/scheduled tribes. Furthermore, practicing Hinduism or other religions, such as Sikhism and Judaism, was positively associated with the CHE incidence as Hindus and other religious groups were 4 and 7.3% more likely vis-a-vis households practicing Islam to face the CHE. The results also underscored the significance of enabling factors in driving the CHE. The evidence indicated an inverse relationship of the CHE with the wealth of households, as richer households were significantly less likely to incur the CHE than their poorer counterparts. The poor, middle, rich, and richest had 11.2, 18.7, 24.1, and 30.5%, respectively, less probability of facing CHE than the poorest household. Analogously, households with government-sponsored insurance cover (6.6%), employer-sponsored cover (10.9%), and private insurance/other covers (12.9%) were less likely to incur CHE vis-a-vis households that are not covered under any financial risk protection scheme. Conversely, households that sought inpatient treatment from private facilities had significantly more likelihood of spending a catastrophic amount on treatment (24.7% for households who sought treatment in a mix of public and private facilities and 32.7% for households who sought treatment in private facilities alone) than those households which sought treatment in just the public hospitals. With respect to the need-based factors, longer duration of hospital stay was associated with more CHE; the probability of incurring CHE was lesser for shorter admission time of fewer than 2 weeks (18.9%), 4–7 days (35.9%), and 3 or fewer days (50.7%) in comparison with the households with longer inpatient days. Finally, the contextual factor of geographical (spatial) location impacted the CHE, as households residing in the regions at higher levels of epidemiological transition level were less likely (7, 4.8, and 6.8% lesser probability for lower-middle, higher-middle, and high epidemiological transition level) to face the CHE on hospital stay as compared to the households residing in the regions having low epidemiolocal level.

**Table 3:** Determinants of the CHE on hospitalization care among households in India.

	Variables	Marginal effects	C.I.
Predisposing factors			
Demographic factors			
Age of household head	Below 25 years^b^		
	25–39 years	−0.027	[−0.087–0.032]
	40–59 years	−0.014	[−0.081–0.053]
	60–75 years	0.028	[−0.060–0.116]
	Above 75 years	0.019	[−0.020–0.057]
Household age composition	With both children and older people^b^		
	With children but no older people	0.011	[−0.068–0.089]
	With older people but no children	0.047***	[0.037–0.057]
	Older people only	0.093***	[0.071–0.116]
	No children or old	0.042**	[0.007–0.077]
Socio-structural factors			
			
Gender of household head	Female^b^		
	Male	−0.021	[−0.029- -0.013]
Size of household	Greater than 10 members^b^		
	5–10 members	0.107***	[0.033–0.179]
	Less than 5 members	0.163***	[0.086–0.240]
Number of widows	Two or more widows^b^		
	One widow	0.007	[−0.090–0.110]
	No widow	0.030	[−0.050–0.110]
Household head education	Illiterate^b^		
	Literate	0.009	[−0.001–0.019]
Principal activity of household	Unpaid worker/Unemployed^b^		
	Self employed	0.025***	[0.024–0.026]
	Casual wage labourer	0.006	[−0.018–0.366]
	Regular/Salaried Wage employee	0.023	[0.009–0.037]
	Pensioner/Retirees	0.022***	[−0.034–0.079]
Social group	Scheduled Tribe/Caste^b^		
	Other backward caste	0.009***	[0.003–0.016]
	Others	0.009	[−0.003–0.021]
Religious affiliation	Islam^b^		
	Hinduism	0.040***	[0.016–0.064]
	Christianity	0.019	[−0.054–0.093]
	Others	0.073***	[0.069–0.077]
Enabling factors			
Expenditure quintile groups	Poorest^b^		
	Poor	−0.112***	[−0.130- -0.094]
	Middle	−0.187***	[−0.194- -0.180]
	Rich	−0.241***	[−0.254- -0.227]
	Richest	−0.305***	[−0.307- -0.303]
Housing conditions	Poor^b^		
	Good	0.007	[−0.011–0.025]
Insurance coverage	No insurance cover^b^		
	Government-sponsored cover	−0.066***	[−0.076- -0.057]
	Employer-sponsored cover	−0.109***	[−0.123- -0.095]
	Private Insurance/Other cover	−0.129***	[−0.141- -0.117]
Type of facility	Only public^b^		
	Both public and private	0.247***	[0.236–0.258]
	Only private	0.327***	[0.322–0.337]
Need factors			
Members with chronic ailments	More than 3 members with chronic ailment^b^		
	2 members with chronic ailment	−0.064	[−0.242–0.114]
	No member with chronic ailment	−0.083	[−0.268–0.102]
Hospitalization episodes	More than five episodes^b^		
	Three-five episodes	−0.063	[−0.488–0.361]
	Two episodes	−0.087	[−0.522–0.347]
	One episode	−0.126	[−0.547–0.295]
Duration of stay in hospitals	More than 2 weeks		
	7–14 days	−0.189***	[−0.204- -0.175]
	4–7 days	−0.359***	[−0.365- -0.353]
	1–3 days	−0.507***	[−0.530- -0.484]
Contextual factors			
Epidemiological transition level	Low epidemiological level^b^		
	Lower-middle epidemiological level	−0.072***	[−0.092- -0.051]
	Higher-middle epidemiological level	−0.048***	[−0.059- -0.037]
	High epidemiological level	−0.068***	[−0.070- -0.066]
Sector	Rural^b^		
	Urban	−0.008	[−0.043–0.026]

Level of significance: ***p < 0.01; **p < 0.05; *p < 0.1. ^b^Reference category.

### 3.5 Decomposition of the inequalities in the CHE on hospitalization care in India

**Figure 4:**
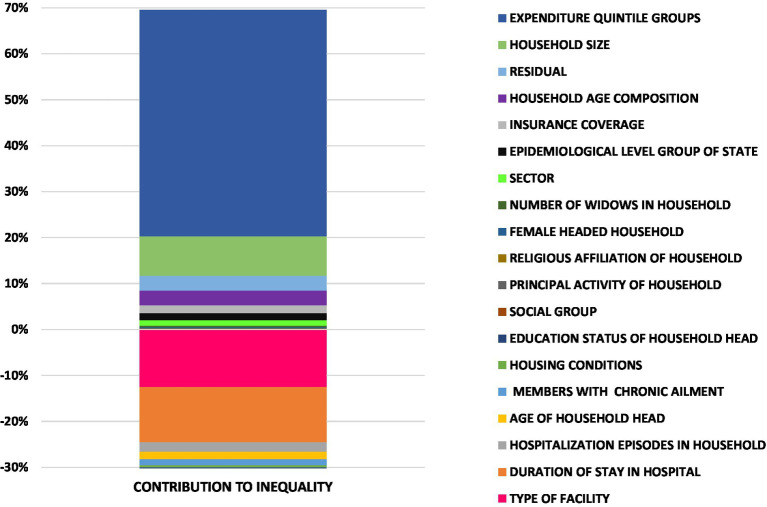
Decomposition analysis of income-related inequalities in CHE on hospitalization.

The results ascertaining the contribution of various determinants in driving the wealth-related inequality in CHE on hospitalization care in India is encapsulated in Table 4, which presents the estimates of coefficients, Erreyger’s concentration indices, absolute contributions (computing the product of elasticity and regressor’s concentration index), and relative contributions (denoting the percentage of inequality in CHE attributable to the inequality in the contributing factor). A positive (negative) value of the absolute contribution of a correlate demonstrates that if the inequality in the CHE was determined by that correlate alone, then it would be concentrated toward the worse-off (better off). The relative contribution of a correlate is computed by dividing the absolute contribution of correlates by total inequality in the outcome variable and multiplying it by 100. The aggregate relative contributions of covariates in driving the inequality are also illustrated in Figure 4. Overall, the relative contribution of need-based variables was exhibited to be negative, connoting that if the CHE were determined by need alone, it would be more concentrated among the poor. Aggregately, the need factors accounted for 35.3% of the unstandardized concentration index, and most of this contribution was attributed to the duration of stay (30.6% of the unstandardized concentration index) in the hospital. However, the inequality push toward the poor was offset to a degree by the effect of the non-need/illegitimate factors. The majority of the inequality in the CHE was driven by illegitimate/non-need factors, with most of the contributions from the enabling factors such as inequality in the wealth of households (expenditure quintiles) and health utilization pattern (facility mix for hospitalization) in conjunction with socio-structural variables such as the size of the household. Additionally, the decomposition results enable the estimation of horizontal inequity, which is obtained by subtracting the absolute need contributions (0.068) from the unstandardized index (−0.19), thus yielding an index value of −0.26.

**Table 4:** Regression coefficients (B), absolute contribution and relative contribution of determinants to income-related inequality in catastrophic health expenditure on hospitalization in India.

	Variables	Coefficient	EI value	Absolute	Relative
Legitimate variables					
Age of household head	Below 25 years^b^				
	25–39 years	−0.016	−0.158	0.002	−0.013
	40–59 years	0.003	0.068	0.000	−0.001
	60–75 years	0.048	0.095	0.004	−0.024
	76–115 years	0.038	−0.253	−0.010	0.052
Household age composition	With both children and older people^b^				
	With children but no older people	0.014	0.030	0.000	−0.002
	With older people but no children	0.039***	0.138	0.005	−0.028
	Older people only	0.129***	−0.088	−0.011	0.060
	No children or old	0.040**	−0.253	−0.010	0.053
Members with chronic ailment	More than 3 members with chronic ailment^b^				
	2 members with chronic ailment	−0.073**	0.137	−0.010	0.052
	No member with chronic ailment	−0.094**	−0.174	0.016	−0.085
Hospitalization episodes in household	More than 5 episodes^b^				
	3–5 episodes	−0.010	0.121	−0.001	0.006
	2 episodes	−0.030	0.075	−0.002	0.012
	One episode	−0.064	−0.214	0.014	−0.071
Duration of stay in hospital	More than 2 weeks^b^				
	8–14 days	−0.251***	0.010	−0.025	0.131
	4–7 days	−0.463***	−0.021	0.010	−0.050
	1–3 days	−0.582***	−0.128	0.074	−0.387
Subtotal (legitimate)				**0.068**	**−0.353**
Illegitimate variables					
Female-headed household	Yes^b^				
	No	−0.020*	0.068	−0.001	0.007
Household size	Greater than 10 members^b^				
	5–10 members	0.085***	0.549	0.047	−0.244
	1–4 members	0.141***	−0.630	−0.089	0.464
Number of widows in household	2 or widows^b^				
	One widow	0.012	0.050	0.001	−0.003
	No widow	0.037	−0.057	−0.002	0.011
Education status of household head	Illiterate^b^				
	Literate	0.014*	0.118	0.002	−0.009
Principal activity of household	Unemployed/unpaid worker^b^				
	Self employed	0.034***	−0.020	−0.001	0.004
	Casual wage laborer	0.005	−0.099	−0.000	0.003
	Regular/salaried wage employee	0.017	0.034	0.001	−0.003
	Pensioner/retirees	0.031**	0.008	0.000	−0.001
Social group	Scheduled tribe/scheduled caste^b^				
	Other backward caste	0.008	−0.010	−0.000	0.000
	Others	0.004	0.148	0.001	−0.003
Religious affiliation of household	Islam^b^				
	Hinduism	0.032***	−0.081	−0.003	0.014
	Christianity	0.019	0.017	0.000	−0.002
	Others	0.067***	0.023	0.002	−0.008
Expenditure quintile groups	Poorest^b^				
	Poor	−0.114***	−0.317	0.036	−0.189
	Middle	−0.190***	0.041	−0.008	0.041
	Rich	−0.245***	0.339	−0.083	0.433
	Richest	−0.311***	0.602	−0.187	0.977
Housing conditions	Poor^b^				
	Good	0.012	0.203	0.002	−0.019
Insurance coverage	No insurance cover^b^				
	Government-sponsored cover	−0.071***	−0.050	0.003	−0.019
	Employer-sponsored cover	−0.100***	0.045	−0.004	0.023
	Private insurance/other cover	−0.152***	0.050	−0.008	0.040
Type of facility	Only public^b^				
	Both public and private	0.240***	0.040	0.009	−0.050
	Only private	0.310***	0.168	0.052	−0.271
Epidemiological level group of state	Low epidemiological level^b^				
	Lower-middle epidemiological level	−0.069***	0.042	−0.003	0.015
	Higher-middle epidemiological level	−0.043***	−0.034	0.001	−0.008
	High epidemiological level	−0.076***	0.079	−0.006	0.031
Sector	Rural^b^				
	Urban	−0.018***	0.320	−0.006	0.031
Subtotal (illegitimate)				**−0.244**	**1.271**
Residual		−0.016	−0.016	**−0.016**	**0.082**
Erreyger’s index (unstandardized)				**−0.19**	**1.000**
Erreyger’s index (standardized)				**−0.26**	**1.000**

Level of significance: ***p < 0.01; **p < 0.05; *p < 0.1. b, beta.

## 4 Discussion and conclusion

Our study revealed significant wealth-related inequalities in the CHE for hospitalization care in India, with a pervasive gap between the poorest and richest income quintiles. The CHE was concentrated more among the poor, with the incidence of CHE being more than twice for the poorest quintile vis-a-vis the richest quintile group. The findings were corroborated by the negative value of the Erreygers concentration index, denoting the inequalities that are disadvantageous to the poor. Furthermore, need-adjusted inequalities also underscored the systemic inequalities (caused by the factors amenable to the policy change) to be concentrated among the poor. Globally, the evidence on the relationship between CHE and socio-economic status has been mixed, and few findings suggest that the better-off experience more CHE in low- and middle-income settings (LMIC) due to the higher propensity of the rich to consume more health services (46). However, our findings were consonant with the studies conducted in other LMIC settings such as Iran (47), China (48), Malawi (49), Columbia (50), and Sub-Saharan Africa (46), where inequality gradients indicated the poor getting afflicted by the CHE disproportionately. The higher incidence of CHE among the poor can be understood by the fact that for households with low income, even a small proportion of healthcare costs can be catastrophic.

The relatively higher incidence of CHE among the poor is pertinent from a policy perspective as it also connotes the intrinsic disparities in healthcare access and finance. India has launched various programs targeted toward the poor to move along the trajectory of Universal Health Coverage (UHC). To achieve the goal of equitable financial risk protection for the marginalized, India launched flagship initiatives such as the National Rural Health Mission (NRHM) in 2005, providing free cost care to the poor and Rashtriya Swasthya Bima Yojana (RSBY) in 2008, covering the poor population with cashless insurance on hospitalization. However, the relatively higher incidence of CHE among the poor alludes to the inefficacy of these programs in providing financial risk protection to the poor. Furthermore, the empirical evidence on the impact of schemes such as RSBY has concurred with its ineffectiveness in reducing the inpatient out-of-pocket spending and catastrophic inpatient spending (51, 52). However, India recently revamped and bolstered these schemes further for expanded coverage by launching the Ayushman Bharat (AB) Program (National Health Protection Mission) for integrated healthcare. The scheme has two components: (a) AB-Pradhan Mantri Jan Arogya Yojana (AB-PMJAY), which provides cashless cover up to INR 5 lakh per family for hospitalization in secondary and tertiary care to over 10 crore poor and vulnerable families; and (b) AB-Health and Wellness Centers (AB-HWCs) providing comprehensive primary and community-based services free of cost to the population. Furthermore, India has launched other initiatives such as free drugs and diagnostics services and financial assistance to patients living below the poverty line for life-threatening diseases under schemes such as Rashtriya Arogya Nidhi (RAN), Health Minister’s Cancer Patient Fund (HMCPF), and Health Minister’s Discretionary Grant (HMDG). Furthermore, affordable medicines and reliable implants for treatment (AMRIT) deendayal outlets have been opened to make available drugs and implants for cardiovascular diseases (CVDs), cancer, and diabetes at discounted prices to patients (53). Although a legion of health initiatives providing free healthcare to different marginalized sections of society have been launched recently, the impact evaluation of these interventions in reducing the burden of OOP on hospitalization among the poor in India needs to be undertaken.

Our findings indicated that members of more than half of the poor households were hospitalized in private facilities with a disproportionately higher incidence of CHE (38.5% in private facilities vis-a-vis 11.5% in public facilities). A myriad of reasons for the preference for private provider(s) in India has been expounded in literature, such as poor readiness and quality of care, higher waiting times, inconvenient facility timings, long distances, absence of healthcare personnel, and lack of acceptability and trust in public providers (54–57). Hence, it is recommended to strengthen the public healthcare system to encompass NCD care (with a disproportionately higher incidence of CHE) (58) and improve the quality of care in terms of infrastructure, equipment, drugs, and diagnostics. A legion of guidelines and standards to ensure the quality of care has been enforced in India, such as Indian Public Health Standards (IPHS), Mera Aspataal (My hospital), and National Quality Assurance Standards (NQAS). However, the non-compliance of quality protocols and standards has hampered the readiness of public health facilities. Thus, the objective periodic monitoring and evaluation of the quality parameters along the continuum of care is suggested to ensure readiness. Concomitantly, surveillance measures such as record keeping, frequent monitoring of employee absence behavior, detection of absence via biometric attendance, and management-oriented punitive action measures for dereliction of duties can be introduced to minimize absenteeism. Simultaneously, to mitigate the low acceptability and poor confidence in public provider, knowledge dissemination, advocacy, and public engagement activities should be promoted at an individual, household, and community and regional level as a confidence-building measure.

Our findings found a legion of factors influencing CHE on hospitalization care. The role of demographic factors was accentuated in the study, and it was found that households comprising only older adult members incur significantly high CHE on hospitalization, which is in tandem with other studies conducted in India (59). Analogously, our estimates revealed that larger size households experience more CHE, which is conflated by other research conducted in LMICs (60, 61). Additionally, other predisposing socio-structural factors, such as affiliation with the marginalized social group and practicing the religion of Hinduism, are associated with higher CHE, which is consonant with the other studies conducted in India (62–64). Although equity has been a primary goal of the flagship programs launched by the Government of India, the related policy discourse has been focused on the praxis of wealth-related inequalities and has precluded other social disparities, such as religion and caste, as a potential axis of healthcare marginalization (65). The multivariate regression estimates also underscored the role of enabling factors such as the absence of insurance coverage and treatment-seeking in private facilities to increase the CHE significantly. The role of these enabling factors, such as the type of health facility and insurance coverage, in influencing the CHE has also been accentuated in many other studies from similar settings (66, 67).

In the LMIC context, the policy discourse has given impetus to the establishment/extension of national/social health insurance in which service providers are paid from designated government funds, which are partly funded through taxes. India via AB-PMJAY provides such insurance coverage for hospitalization to the poor and vulnerable; however, evidence from rural India suggests that around one-fourth of the eligible participants are still unaware of the AB-PMJAY scheme; moreover, the level of utilization of the scheme has been found to be abysmally low at 1.3% (68). The low level of utilization can be explained via complex enrollment or reimbursement process, which acts as a significant barrier to take up. The findings on PMJAY in India also suggest that this scheme shifted the use of health facilities from public providers to privately empaneled hospitals where the cost of care is higher (69). Thus, a gamut of strategies can be employed to increase the penetration and uptake of Public Funded Health Insurance (PHFI) schemes in India, such as an increase in the awareness of benefits and community engagement via appropriate training for competencies of the community health workers, such as Accredited social health activists (ASHA) and Anganwadi workers (AWW); easing the process of enrollment and reimbursement and streamlining other hospital-based processes for effective implementation of the scheme (70) and establishing a robust referral linkage between the primary healthcare facilities with secondary and tertiary hospitals with the help of digital interventions and infrastructure. However, in regions where the institutional capacity to organize mandatory nationwide risk-pooling is weak, community-based health insurance schemes can be effective in protecting poor households from unpredictably high medical expenses (31).

The findings also demonstrated the role of contextual factors such as the region in influencing the CHE as the households belonging to the states with higher levels of the epidemiological transition level (defined based on the ratio of disability-adjusted life years and computed as the sum of years of potential life lost due to the premature mortality and the years of productive life lost due to disability from communicable disease to those from non-communicable and injuries combined) incurred lesser CHE as compared to their counterparts residing in the states at a lower level of ETL. These inter-region heterogeneities can be explained by the inverse relationship between the epidemiological transition ratio and socio-economic development of the states (71). A higher burden of CHE on the states with a lower level of epidemiological transition is a pertinent finding from the policy perspective as these states are associated with the lower *per capita* expenditure on healthcare, thus lacking financial risk protection vis-a-vis other states. Thus, there is a need to increase public spending on healthcare to reach the targeted level of 4% of GDP by 2025. However, realistically, the state governments can set a target to allocate at least 2.5% of the state’s gross domestic product (SGDP) to healthcare, which is the recommended level by the World Health Organization (WHO). It is further suggested that the government explore new and innovative financing mechanisms to generate the fiscal space, such as the public–private partnership to fund the sector; simultaneously, other fiscal space measures, such as the collection of health-specific tax, goods, and services tax reform, higher excise duty on tobacco products, tax administration reform and direct beneficiary transfer of health services could be employed as the alternative revenue mobilization channels for fiscal space in health (72).

The decomposition analysis revealed that the contribution of non-need/illegitimate factors in driving the inequality was relatively high vis-à-vis need/legitimate factors, as most of the inequality in CHE was driven by the non-need factors amenable to the policy change. Most of the unfair inequalities arose from socio-structural factors such as the size of the household and enabling factors such as income (expenditure) and type of facility (public or private) utilized. The relative contribution of these determinants in influencing inequalities in CHE is found in other LMICs. A study on the decomposition of inequalities in CHE in Iran (47) demonstrated that most of the illegitimate inequalities emanated from household economic status (64%), followed by household size (40%). Other studies in China have also accounted for household size as the largest contributor to CHE inequality (73, 74). Furthermore, evidence from Sierra Leonne suggested that the distributional effect of the type of facility significantly impacted the inequalities in the CHE (75). Thus, from the policy perspective, it is imperative to invest more in public health facilities, providing significant financial risk protection to the poor. From the Indian perspective, the burden of CHE was found to be disproportionately higher for the poor and middle-population groups as well. Thus, it is suggested that the state and central governments expand the PFHI coverage to the missing middle population as well.

The study has a few caveats due to the nature of the dataset and the methodological approach. *First*, the same weights are assigned to the catastrophic payments incurred by poor and non-poor households and, thus, ignore the differentials in the opportunity cost in the health spending between rich and poor, thereby rendering the measure non-normative, which does not allow for distributional sensitivity. *Second*, health expenditures are not adjusted for coping mechanisms such as distressed financing or adjustment in the consumption pattern to pay for the health expenditure, thus understating CHE. *Third*, the data on expenditure used in the survey is self-reported and is susceptible to recall and information bias. *Fourth,* in the multivariate regression, the information on outcome measures and covariates was collected concurrently due to the cross-sectional design; thus, associations rather than causal relationships are defined in the study. *Fifth,* the information on self-reported monthly household consumer expenditure is a one-shot open-ended with no parallel validation, and thus can lead to the underestimation of the household’s income.

## Data availability statement

The original contributions presented in the study are included in the article/supplementary material, further inquiries can be directed to the corresponding author.

## Author contributions

SS: Conceptualization, Data curation, Formal analysis, Funding acquisition, Investigation, Methodology, Project administration, Resources, Supervision, Validation, Visualization, Writing – original draft, Writing – review & editing. VV: Data curation, Formal analysis, Methodology, Project administration, Software, Supervision, Validation, Visualization, Writing – original draft, Writing – review & editing. PG: Data curation, Formal analysis, Investigation, Methodology, Visualization, Writing – review & editing. MA: Data curation, Formal analysis, Investigation, Methodology, Resources, Software, Validation, Writing – review & editing.
